# Identification of a novel combination treatment strategy in clear cell renal cell carcinoma stem cells with shikonin and ipilimumab

**DOI:** 10.3389/fimmu.2023.1186388

**Published:** 2023-08-09

**Authors:** Chen Lyu, Birgit Stadlbauer, Lili Wang, Alexander Buchner, Heike Pohla

**Affiliations:** ^1^ Tumor Immunology Laboratory, LIFE Center, LMU Klinikum, University Munich, Planegg, Germany; ^2^ Department of Urology, LMU Klinikum, University Munich, Munich, Germany; ^3^ Department of Radiology, First Affiliated Hospital, Zhejiang University School of Medicine, Hangzhou, China

**Keywords:** renal cell carcinoma, immune checkpoint inhibitor (ICI), phytochemical, immunotherapy, cancer stem cells (CSCs), tumor microenvironment (TME), tumor infiltrating immune cell (TIC)

## Abstract

**Background:**

Management of clear cell renal cell carcinoma (ccRCC) has changed rapidly in recent years with the advent of immune checkpoint inhibitors (ICIs). However, only a limited number of patients can sustainably respond to immune checkpoint inhibitors and many patients develop resistance to therapy, creating an additional need for therapeutic strategies to improve the efficacy of systemic therapies.

**Methods:**

Binding probability and target genes prediction using online databases, invasion, migration, and apoptosis assays as well as the inhibition of cancer stem cells (CSCs) markers in ccRCC cell lines were used to select the most promising phytochemicals (PTCs). Mixed lymphocyte tumor cell culture (MLTC) system and flow cytometry were performed to confirm the potential combination strategy. The potential immunotherapeutic targets and novel CSC markers were identified via the NanoString analysis. The mRNA and protein expression, immune signatures as well as survival characteristics of the marker in ccRCC were analyzed via bioinformation analysis.

**Results:**

Shikonin was selected as the most promising beneficial combination partner among 11 PTCs for ipilimumab for the treatment of ccRCC patients due to its strong inhibitory effect on CSCs, the significant reduction of FoxP3^+^ Treg cells in peripheral blood mononuclear cells (PBMCs) of patients and activation of the endogenous effector CD3^+^CD8^+^ and CD3^+^CD4^+^ T cells in response to the recognition of tumor specific antigens. Based on NanoString analysis VCAM1, CXCL1 and IL8 were explored as potential immunotherapeutic targets and novel CSC markers in ccRCC. The expression of VCAM1 was higher in the tumor tissue both at mRNA and protein levels in ccRCC compared with normal tissue, and was significantly positively correlated with immune signatures and survival characteristics in ccRCC patients.

**Conclusion:**

We propose that a combination of shikonin and ipilimumab could be a promising treatment strategy and VCAM1 a novel immunotherapeutic target for the treatment of ccRCC.

## 1 Introduction

Renal cell carcinoma (RCC) is the most common type of renal malignancy, of which clear cell renal cell carcinoma (ccRCC) or kidney renal clear cell carcinoma (KIRC) accounts for about 70-85% of cases ranging from an indolent evolution to a rapid and widespread progression. More than 30% of ccRCC patients are metastatic at the time of the diagnosis, and nearly 30% will progress to metastasis during the course of follow-up (1, 2). Recently, the management of metastatic ccRCC has been revolutionized by the advent of immune checkpoint inhibitors (ICIs) replacing or being added to treatments with tyrosine kinase inhibitors (TKIs) alone, and each combination regimen is considered highly effective, with objective response rates ranging from 42% to 71% (3–6). Meanwhile, dual ICI treatment, combining nivolumab and ipilimumab, has also shown a survival benefit and reduced the risk of death by 32%. In 2018, this therapy was approved for previously untreated RCC patients with intermediate and low risk (7). Furthermore, in a recent phase 3 clinical trial (NCT03937219), the combination of cabozantinib, nivolumab, and ipilimumab demonstrated statistically significant improvements in progression-free survival (PFS) among 428 participants. However, it is worth noting that the treatment also resulted in a high rate of grade 3/4 adverse events (AEs), with nearly 80% of participants experiencing these severe AEs (8). Despite significant improvements in systemic therapies for ccRCC, only a few patients have achieved a durable clinical response, with the median PFS ranging from 11.6 to 15.4 months, even with first-line treatment (9) followed by the therapy-resistance. Additional therapeutic strategies to improve the efficacy of systemic therapies are therefore urgently needed, especially in patients with limited disease burden.

Targeting cancer stem cells (CSCs), a small population of cancer cells in the tumor microenvironment (TME), has been suggested as the key to successful treatment against the increased relapse rate of cancers toward current chemo- or radiotherapy (10). Emerging evidence has indicated that renal tumorigenesis and RCC treatment resistance may originate from renal CSCs with tumor-initiating capacity (11, 12). So far, many studies have tried to establish unique biomarkers to identify CSC populations in RCC. Consequently, several markers were found to be specifically expressed in CSCs and cancer stem-like cells derived from RCC such as CD105, ALDH1, OCT4, CD133, and CXCR4 which have the ability to play multiple functional roles in regulating stem cell function (13). For example, CXCR4^+^ cells derived from several RCC cell lines exhibit resistance to therapy (TKIs) and enhanced capability to form spheres *in vitro* and tumors *in vivo* compared to CXCR4^–^cells (14). Furthermore, several CSC markers such as EZH2, OCT4 and NANOG could be considered as novel independent prognostic predictors in patients with renal cancer (15, 16). Therefore, the identification of a specific CSC marker for RCC that either initiate or maintain tumorigenesis is of most importance for understanding tumor biology and in the development of novel therapies.

To target CSCs, phytochemicals have been proposed due to their economical nature, less immunological response and relatively low side effects (17, 18). Moreover, merging evidence showed that several phytochemicals such as curcumin, piperine, berbamine, shikonin, genistein as well as the whole extract of some plants are able to kill CSCs (19, 20). For instance, epigallocatechin-3-gallate (EGCG) as an active compound in green tea is involved in several ongoing alone or combination clinical trials with cisplatin and oxaliplatin, because of the potential to suppress cancer stemness and tumorigenicity and its ability to improve the efficacy of conventional drugs in several types of cancers including RCC (21–23). The usage of phytochemicals is likely to be a potential treatment strategy for eradicating cancer through the elimination of CSCs. This is a milestone in the improvement of cancer treatment because the synthetic anticancer drugs that are currently used are often highly toxic to healthy organs and weaken the patient’s immune system. Therefore, more clinical trials could be released to improve the outcomes of these patients through the usage of a combination therapy with phytochemicals and immunotherapy or other more efficient systemic therapies.

## 2 Materials and methods

### 2.1 Prediction of binding probability and intersection genes

The two-dimensional (2D) and three-dimensional (3D) structure of phytochemicals was acquired from the PubChem database (https://pubchem.ncbi.nlm.nih.gov/). The three-dimensional structures of the target proteins were downloaded from protein data bank (https://www.rcsb.org/) with the PDB ID: 7KEZ (VEGF), 3HN4 (HGF), 3MJG (PDGF), 3FUB (GDNF) and 4DRH (mTOR) respectively. The binding probability based on the predicted structures between target proteins and phytochemicals was evaluated by the online platform Kdeep (https://playmolecule.com/Kdeep/). Kdeep is a protein-ligand affinity predictor based on Deep Convolutional Neural Networks (DCNNs). The SDF file of the 3D structure was uploaded to the PharmMapper database (http://www.lilab-ecust.cn/pharmmapper/) for potential target gene prediction.

ccRCC (KIRC, n=539) immune-related genes were extracted from The Cancer Genome Atlas database (TCGA) database (https://portal.gdc.cancer.gov/). The intersection of ccRCC immune genes and phytochemicals’ potential target genes were obtained using a Venn diagram.

### 2.2 Cell culture

SKRC-17 (kind gift from J. Vissers, Nijmegen), and RCC-53 (derived from a patient with stage IV disease (pT2N1MxG2-3)) were grown in RPMI1640 supplemented with 10% fetal calf serum (FCS “Gold Plus”, Bio & Sell GmbH, Feucht, Germany), 1% minimal essential medium, 1 mM sodium pyruvate, and 2 mM L-glutamine (Invitrogen, Life Technologies GmbH, Darmstadt, Germany) under the condition at 37°C in a humidified incubator with 5% CO_2_. The corresponding CSCs were generated using CSC medium containing DMEM/F12 medium supplemented with 2% B-27 (Invitrogen), 10 ng/ml epidermal growth factor (EGF, Sigma Aldrich Chemie GmbH, Taufkirchen, Germany), and 10 ng/ml basic fibroblast growth factor (bFGF, Sigma Aldrich).

### 2.3 Sphere formation assay

All the CSCs were generated by the sphere-forming assay in CSC specific medium. Initially, SKRC-17 and RCC-53 were harvested using 3-5 ml Accutase cell detach solution (Life Technologies, Thermo Fisher Scientific, Waltham, MA, USA) and incubated for 8-10 minutes at 37°C. Then, 3-10 × 10^5^ cells were seeded in 75 cm^2^ ultra-low attachment flasks (Corning, New York, NY, USA) and cultured with 10 ml CSC specific medium for 7 days.

### 2.4 Quantitative reverse transcription PCR (RT-qPCR)

Total RNA was extracted from cells using the RNeasy Mini-Kit (Qiagen, Hilden, Germany) based on the manufacturer’s instructions. cDNA was synthesized according to the kit instructions (Reverse Transcription System, Promega GmbH, Mannheim, Germany). The real-time PCR procedure was performed using the LightCycler® 96 (Roche) and the DNA Green Master kit (Roche, Penzberg, Germany). The reaction started with 95°C for 10 minutes, followed by 40 cycles of denaturation at 95°C for 10 seconds, annealing at 56-62°C for 10 seconds, and extension at 72°C for 10 seconds. Data were analyzed by the LightCycler® 96 software version 1.1. The relative expression analysis was carried out by the 2^-ΔΔCt^ method. The transcription level of *GAPDH* and *ACTB* was used as an internal control, and the primers of *VCAM1* (GeneGlobe ID: PPH00623E-200), *CXCL1* (GeneGlobe ID: PPH00696C-200) and *IL8* (GeneGlobe ID: PPH00568A-200) were obtained from the RT^2^ qPCR Primer Assays (Qiagen, Hilden, Germany). The other primers were listed in **Table 1**.

**Table 1:** Primers used for RT-qPCR.

Transcript	Primer	Sequence (5’-3’)	product size (bp)
*GAPDH*	GAPDH-F	CATGGGTGTGAACCATGA	104
	GAPDH-R	TGTCATGGATGACCTTGG	
*ACTB*	ACTB-F	CTGCCCTGAGGCACTC	197
	ACTB-R	GTGCCAGGGCAGTGAT	
*ABCA13*	ABCA13-f	AGGAGTGTGAGGCTCTTTGC	207
	ABCA13-r	TCAGGTGCTGTCCCTTGAAC	
*ABCB1*	ABCB1-f	GGAGGCCAACATACATGCCT	205
	ABCB1-r	CAGGGCTTCTTGGACAACCT	
*ABCG2*	ABCG2-f	CATCAACTTTCCGGGGGTGA	266
	ABCG2-r	CACTGGTTGGTCGTCAGGAA	
*ALDH1A1*	ALDH1A1-f	TGTTAGCTGATGCCGACTTG	154
	ALDH1A1-r	TTCTTAGCCCGCTCAACACT	
*ALDH1A3*	ALDH1A3-f	GAGGAGATTTTCGGGCCAGT	186
	ALDH1A3-r	GAGGGCGTTGTAGCAGTTGA	
*ALDH3A1*	ALDH1A3-f	GCAGACCTGCACAAGAATGA	186
	ALDH1A3-r	TGTAGAGCTCGTCCTGCTGA	
*CD105*	ENG-f	TCACCACAGCGGAAAAAGGT	141
	ENG-r	GGACACTCTGACCTGCACAA	
*CD133*	PROM1-f	TTGCGGTAAAACTGGCTAAG	155
	PROM1-r	TGGGCTTGTCATAACAGGAT	
*CXCR4*	CXCR4-f	TGGGTGGTTGTGTTCCAGTTT	80
	CXCR4-r	ATGCAATAGCAGGACAGGATG	
*DAB2IP*	DAB2IP-f	TGTCGCCCTCACTCTTCAAC	225
	DAB2IP-r	CGGCTGTATTGGAGAGGGTC	
*DNMT1*	DNMT1-f	GGCAGACCATCAGGCATTCT	220
	DNMT1-r	ACCATGTCCTTGCAGGCTTT	
*EZH2*	hEZH2-f	AGGACGGCTCCTCTAACCAT	179
	EZH2-r	CTTGGTGTTGCACTGTGCTT	
*KLF4*	KLF4-f	TCCCATCTTTCTCCACGTTC	239
	KLF4-r	GGTCTCTCTCCGAGGTAGGG	
*LIN28A*	LIN28A-f	TTCGGCTTCCTGTCCATGAC	124
	LIN28A-r	CCACTGCCTCACCCTCCTT	
*MTGR1*	MTGR1-f	CCTCCTACCCTGAATGGTGC	214
	MTGR1-r	GTGCAAGAACAAGAGTCCGC	
*NANOG*	NANOG-f	TGTGTTCTCTTCCACCCAGC	205
	NANOG-r	CTTCTGCGTCACACCATTGC	
*POU5F1*	POU5F1-f	CCCTGGGGGTTCTATTTGGG	231
	POU5F1-r	TCTCCAGGTTGCCTCTCACT	
*SALL4*	SALL4-f	GCTCTGTTAGGTACGGACGG	96
	SALL4-r	CTGGTTCCACACAACAGGGT	
*SOX2*	SOX-2-f	CATCACCCACAGCAAATGAC	258
	SOX-2-r	GCAAACTTCCTGCAAAGCTC	

### 2.5 Apoptosis assay

Apoptosis assay was executed by flow cytometry using Annexin V and 7-aminoactinomycin D (both from BD Biosciences). A total of 4×10^5^ cells were seeded in 25 cm^2^ flasks and cultured with or without phytochemicals. After five days cells were harvested and resuspended in Annexin V binding buffer, stained with APC-conjugated Annexin V and 7-aminoactinomycin D (7-AAD) and incubated for 15 minutes in the dark at room temperature. Samples were measured within on hour using the FACSCalibur (Becton Dickinson, San Jose, CA, USA). For each sample, a minimum of 1×10^4^ cells were recorded. Data acquisition was done using BD CellQuest software (version 4.0.2) and analyzed using FlowJo (version 9.9.5; Tree Star Inc., Ashland, OR, USA).

### 2.6 Drug sensitivity assay

CellTiter Blue Cell Viability Assay (Promega, Madison, USA) was used to determine IC50 of ICIs. 1-5 × 10^3^ cells per well were seeded in 96-well plates and incubated overnight at 37°C and 5% CO_2_. Then, the culture medium was exchanged with or without ICIs on the following day. After 48h, a volume of 20 µl CellTiter Blue Solution was added to each well, and the plates were incubated for two hours at 37°C and 5% CO_2_. Finally, the data were collected using the FLUOstar OPTIMA microplate reader (BMG LABTECH, Ortenberg, Germany) at 560 (20) nm excitation and 590 (10) nm emission. The wells without cells were again used as background controls, while the wells with cells but without treatment were the control group. The OPTIMA software version 2.0 was utilized to collect and analyze the data, while the IC50 was calculated using the logit regression model.

### 2.7 Mixed lymphocyte tumor cell culture (MLTC)

After 7 days incubation CSCs were harvested, dissociated, treated with or without shikonin, seeded into a 24-well plate with 5 × 10^4^ cells per well and incubated overnight with CTL Test medium (Cellular Technology Ltd. Europe, Bonn, Germany). Next day, PBMCs were thawed and washed in CTL wash supplemented medium (45 ml RPMI 1640 medium, 5 ml CTL Wash (Cellular Technology Ltd. Europe)) and 50 U/ml Benzonase (Novagen Merck Biosciences, Darmstadt, Germany). PBMCs and ICIs were added and incubated for five days together with the CSCs with addition of a final concentration of 50 U/ml IL-2(Proleukin, Novartis, Basel, Switzerland) after 48 hours.

### 2.8 Flow cytometry

Tumor cells were diluted to 1-2×10^6^ cells and incubated with the LIVE/DEAD™ Fixable Blue Dead Cell Stain (Molecular Probes, Life Technologies, ThermoFisher Scientific, Waltham, MA, USA) for 30 min at room temperature, then washed with PBS twice. To stain with the directly labeled monoclonal antibodies, cells with antibodies were incubated for 30 min at 4°C in the dark, then washed with PBS twice. For intracellular staining with the FoxP3 antibody, the FoxP3/Transcription Factor Staining Buffer Set (eBiosciences) was used, and staining was done for 60 min at 4°C in the dark and washed with the Perm buffer twice.

For lymphocyte staining the following directly conjugated mouse monoclonal antibodies were purchased from BD Biosciences: CD3 (clone UCHT1, FITC), CD4 (clone SK3, PE-Cy7), CD8 (clone RPA-T8, APC), CD25 (clone M-A251, PerCP-Cy5.5), CD127 (clone hIL-7R-M21, PE), respectively. For Treg analysis, the monoclonal antibody FoxP3 (clone PCH101, eFluor450; eBiosciences, Frankfurt, Germany) was used.

All measurements were accomplished using the LSRII flow cytometer (BD Biosciences). Data analyses were performed by FlowJo software (version 9.9.0; Tree Star Inc., Ashland, OR, USA).

### 2.9 Migration assay

A scratch wound healing assay was performed with 24-well µ-plates containing small 2-well silicone inserts per well, which included a cell-free gap of 500 μm as space for the cells to migrate (ibidi GmbH, Martinsried, Germany). 70 µl of a cell suspension of 4 x 10^5^ cells/ml culture medium were added to each well of the small insert and incubated at 37°C and 5% CO_2_ for at least 24 hours until a confluent cell monolayer was achieved. Then the inserts were removed and the cell layer was washed with PBS to remove cell debris and non-attached cells. After addition of new culture medium with or without the phytochemicals at different concentrations the plate was incubated for another 15 hours and pictures were taken at several time points (0, 3, 6, 9, 12, and 15 h). The percentage of covered area of the gap was assessed and analyzed by the Automated Cellular Analysis System (ACAS, MetaVì Labs, Bottrop, Germany) based on the FastTrack AI image analysis algorithms.

### 2.10 Invasion assay

Invasion assay was performed using the Boyden Chamber system with transwell inserts (8.0 µm pores; Falcon, Corning, New York, NY, USA) in 24-well plates coated with growth factor reduced Matrigel Basement Matrix (Corning; 30 µg/100 µl/insert). 30,000 cells were seeded in 250 µl serum-free medium with or without phytochemicals onto the Matrigel-coated insert, and the lower chamber was filled with 750 µl DMEM with 10% FCS. The cells were incubated at 37°C and 5% CO_2_ for 48 hours. Then, the upper surface of the transwell membrane was gently wiped with a moistened cotton swab to remove Matrigel with not migrated cells. Invaded cells were fixed with 4% paraformaldehyde for 5 minutes, stained with 1% crystal violet for 1 minute, washed twice with water, and dried on a paper towel at room temperature. Finally, pictures were taken with a digital camera (three fields per insert), and cells were counted using the Fiji Image J software.

### 2.11 NanoString analysis

Gene expression analysis was performed using the human nCounter® PanCancer-Immune-Profiling-Panel-(Human) (NanoString Technologies, Seattle, WA, USA) according to the manufacturer’s protocol with 100 ng of total RNA from corresponding cells. Downstream analysis in terms of heatmaps and volcano plot were performed using nSolver 4.0.

### 2.12 Target gene prediction

The three-dimensional (3D) structure of shikonin was acquired from the PubChem database (https://pubchem.ncbi.nlm.nih.gov/). The SDF file of the 3D structure was uploaded to the PharmMapper database (http://www.lilab-ecust.cn/pharmmapper/) for potential target gene prediction. Top 100 of CTLA-4-related genes based on the ccRCC samples of the TCGA database were obtained from GEPIA (http://gepia.cancer-pku.cn/index.html). Protein-protein interaction (PPI) network was constructed by STRING database with a confidence score > 0.4, followed by reconstruction with Cytoscape version 3.6.1. Nodes with confidence of interactive relationship larger than 0.95 were used for building the network.

### 2.13 Gene expression in tumor and normal tissue

Transcriptome RNA-seq data of 611 ccRCC cases (KIRC, normal samples: 72 cases; tumor samples: 539 cases) were downloaded from the TCGA database. Significantly differently expressed genes in ccRCC samples were displayed in a volcano plot from R software’s package limma (https://www.R-project.org).

The UALCAN portal (http://ualcan.path.uab.edu/analysis-prot.html) was used to conduct mRNA and protein expression analysis of the CPTAC (Clinical Proteomic Tumor Analysis Consortium) dataset [49]. Herein, we explored the expression level of total protein of VCAM1 between primary tumor and normal tissues, respectively, by entering “VCAM1”.

We used the “Expression Analysis-Box Plots” module of the GEPIA2 (Gene Expression Profiling Interactive Analysis, version 2) web server (http://gepia2.cancer-pku.cn/#analysis) to obtain box plots of the expression differences between these tumor tissues and the corresponding normal tissues of the GTEx (Genotype-Tissue Expression) database [48], upon the settings *p*-value cutoff = 0.01, log2FC (fold change) cutoff =1, and “Match TCGA normal and GTEx data”.

Immunohistochemistry pictures of VCAM1 were downloaded from the human protein atlas (www.proteinatlas.org) to confirm the protein expression.

### 2.14 Prognosis analysis

Survival analysis of ccRCC patients was obtained from the website-GEPIA (http://gepia.cancer-pku.cn/index.html). Kaplan–Meier method was used to calculate the cumulative event (death) rate, according to the duration from the date of operation to the date of death as the outcome variable. Survival curves stratified by risk factors were operated by log-rank test, the *p*-values < 0.05 were considered to show the statistical significance. The median group cutoff was median survival times of the groups. Multivariable COX regression was shown in the forest plot, which was conducted by the R statistical software language with package survival and survminer.

### 2.15 Tumor immunological signatures

CIBERSORT computational method was applied for estimating the profile of tumor-infiltrating cell (TIC) subtypes in ccRCC tumor samples, followed by quality filtering resulting in 539 tumor samples with *p* < 0.05 for display in a plot, which was conducted by R software.

TIMER (https://cistrome.shinyapps.io/timer/) was used to determine the correlation with the infiltration of the immune cells (neutrophils, macrophages, dendritic cells, B cells, and CD4/CD8 T cells) in the TME upon the module of somatic copy number alteration (SCNA) module in ccRCC.

The data from UCSC database (https://xenabrowser.net/) was used to perform the ESTIMATE score and immunophenoscore (IPS) analysis. R package named ESTIMATE was operated to conduct the stromal, immune, and ESTIMATE scores in ccRCC; package-IOBR was utilized to value the MHC (Antigen processing), CP (checkpoint), EC (effector cells), SC (suppressor cells), average Z-score (AZ) and immunophenoscore (IPS) in ccRCC.

### 2.16 VCAM1-related genes

The correlation between 60 genes for immune checkpoint genes as well as 44 genes for RNA modifcations modulate genes and VCAM1 were studied based on the UCSC database (https://xenabrowser.net/). The correlations were calculated by the Pearson correlation coefficient.

GeneMANIA online database tool (http://www.genemania.org) was applied for VCAM1-related gene analysis and its protein-protein interaction (PPI) analysis, which includes physical interaction, co-expression, co-localization, gene enrichment analysis, genetic interaction and website prediction.

### 2.17 Gene Set Enrichment Analysis (GSEA)

KEGG gene sets (c2.cp.kegg.v7.0.symbols.gmt) were downloaded from the Molecular Signatures Database (https://www.gsea-msigdb.org/gsea/msigdb/genesets.jsp) as target sets with which GSEA was performed by the software gsea-4.1. For analysis, gene set permutations were done 1000 times to obtain a normalized enrichment score, which was used for sorting pathway enrichment. NOM *p* < 0.05 and false discovery rate (FDR) *q* < 0.06 were considered as significant.

## 3 Results

### 3.1 Prediction of potential RCC-targeting phytochemicals

**Figure 1:**
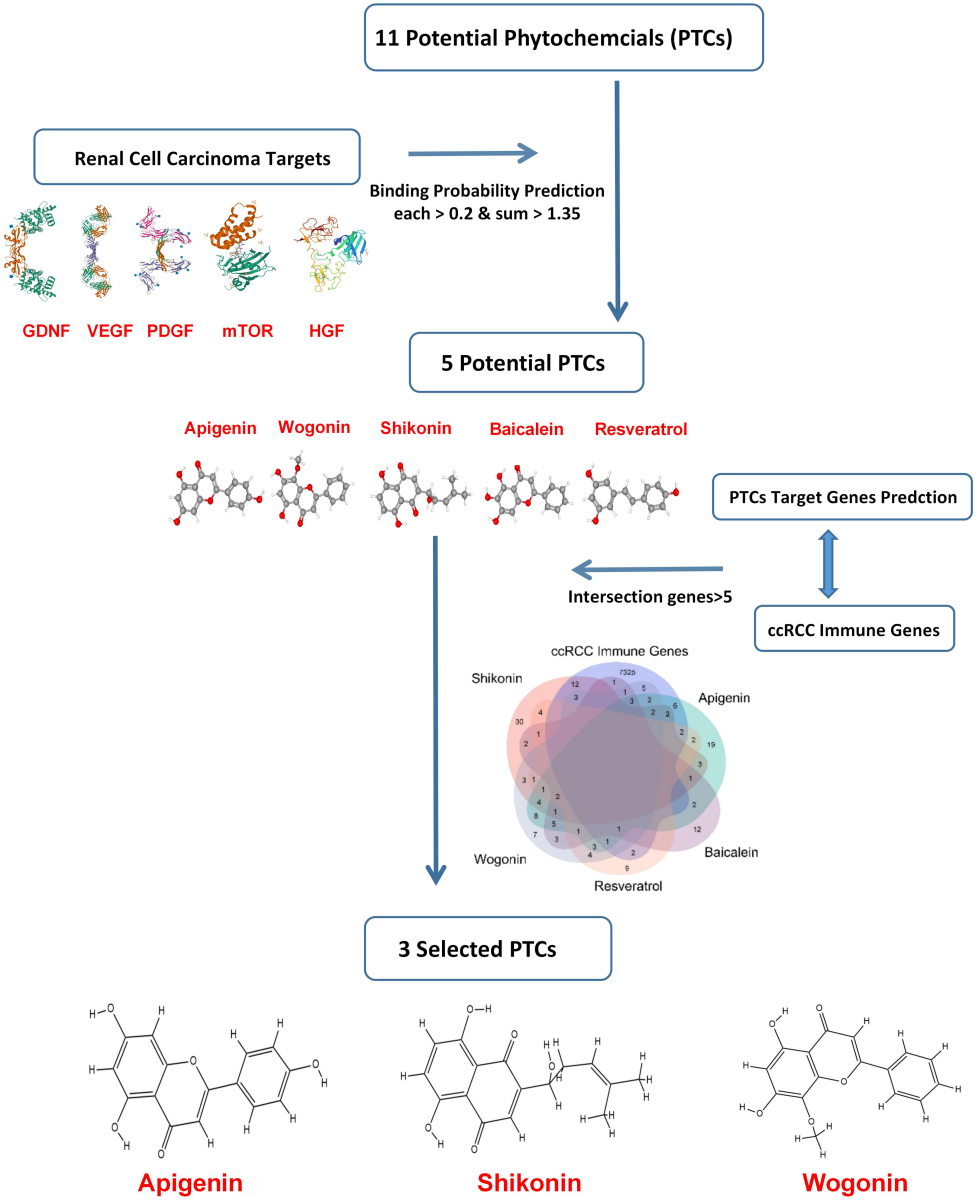
Scheme for selection of phytochemicals targeting RCC.

Based on previous research, the RCC therapeutic target proteins taken for the study were VEGF (vascular endothelial growth factor), HGF (hepatocyte growth factor), PDGF (platelet-derived growth factor), GDNF (glial cell derived neurotrophic factor), and mTOR (mammalian target of rapamycin) (24–27). Then the binding probability between 11 potential RCC-targeted phytochemicals and five RCC therapeutic target proteins was predicted, and only the binding probability value for each protein target above 0.2 and the sum value for five target proteins above 1.35 could be moved to the next selection. Finally, taking the intersection of five phytochemicals’ potential target genes and ccRCC immune-related genes, three phytochemicals: shikonin, apigenin, and wogonin had more than five of the intersection genes and were decided for further testing in this study (**Figure 1**).

### 3.2 Shikonin, apigenin and wogonin enhance the apoptosis rate and inhibit migration and invasion of adherent cell lines and CSCs

**Figure 2:**
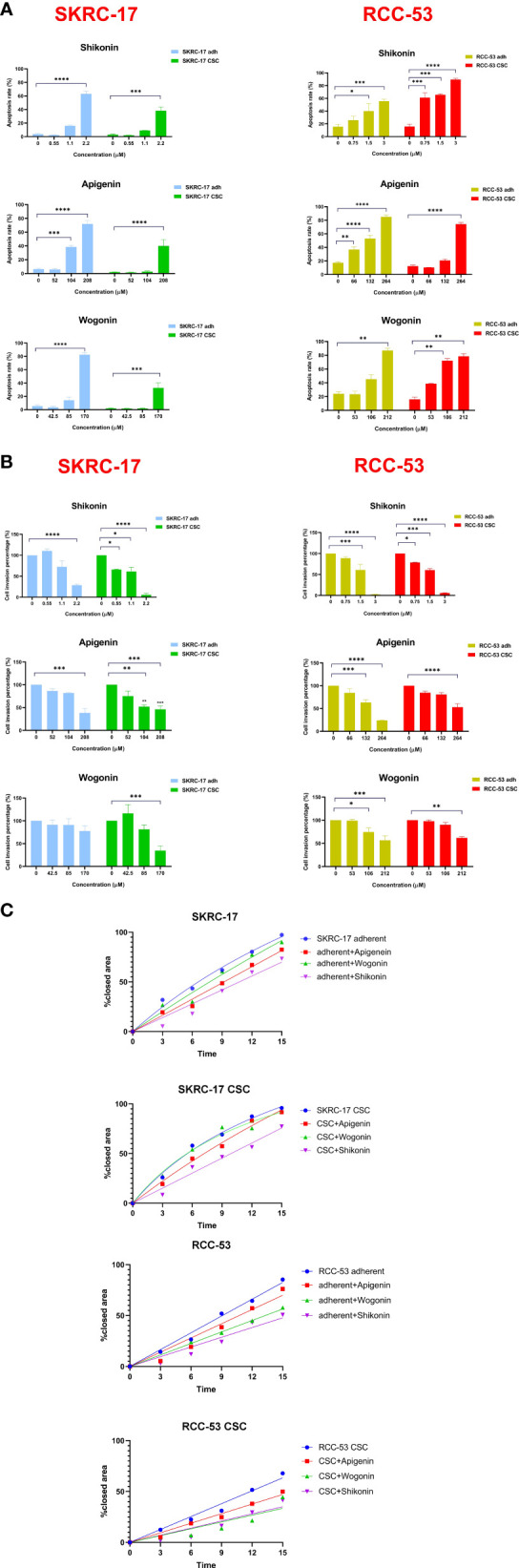
Selection of the three potential PTCs targeting RCC cell lines. **(A)** Apoptosis analysis, SKRC-17 and RCC-53 cell lines were treated with different concentrations of shikonin, apigenin and wogonin (0.5 x IC50, 1 x IC50, and 2 x IC50) stained with APC-conjugated Annexin V and 7-AAD and measured by flow cytometry. **(B)** For invasion, the Boyden chamber assay was used. Cells were again treated with PTCs (0.5 x IC50, 1 x IC50, 2 x IC50) for 48 (h) The cells were counted via Fiji ImageJ software. **(C)** Migration was examined using the wound healing assay. The cells were treated with PTCs (0.5 x IC50) and pictures were taken at the time points 0 h, 3 h, 6 h, 9 h, 12 h, and 15 (h) The data were analyzed with the web-based Automated Cellular Analysis System using FastTrack AI image analysis algorithms. (n = 3, *p < 0.05, **p < 0.01, ***p < 0.001, ****p < 0.0001).

SKRC-17 and RCC-53 and the corresponding CSCs were treated with increasing doses of the PTCs according to the IC50 values tested in our former study (0.5 × IC50, IC50, and 2 × IC50) for 5 days, and the results demonstrate a dose-dependent effect of the PTCs on the apoptosis (**Figure 2A**; **Supplementary Figure S1**) (15). The data showed that SKRC-17 CSCs and RCC-53 CSCs were much less sensitive to the phytochemicals than the corresponding adherent cells in apigenin and wogonin groups. It supported the point that cancer stem cells were a special cell population like drug-resistant cells. Furthermore, since migration and invasion ability are two important biological characteristics, we evaluated the effect of phytochemicals on the migration and invasion of SKRC-17, RCC-53 and their corresponding sphere cells. For the invasion assay, the cells were treated again with different concentrations of the PTCs (0.5 × IC50, 1 × IC50, and 2 × IC50) for 48 h. The number of invaded cells was significantly decreased in the adherent cell lines and even in the corresponding CSCs for the apigenin and shikonin group (**Figure 2B**; **Supplementary Figure S2**). For the migration assay the cells were treated with a dose of 0.5 x IC50. Pictures were taken at different time points (0 h, 3 h, 6 h, 9 h, 12 h and 15 h). The percentage of covered area of the gap was assessed and analyzed by the Automated Cellular Analysis System based on the FastTrack AI image analysis algorithms (**Figure 2C**; **Supplementary Figure S3**). Shikonin had the highest potential and was able to inhibit both the migration and invasion remarkably in the cell’s lines and corresponding CSCs.

### 3.3 Phytochemicals regulate the expression of CSC biomarkers

**Figure 3:**
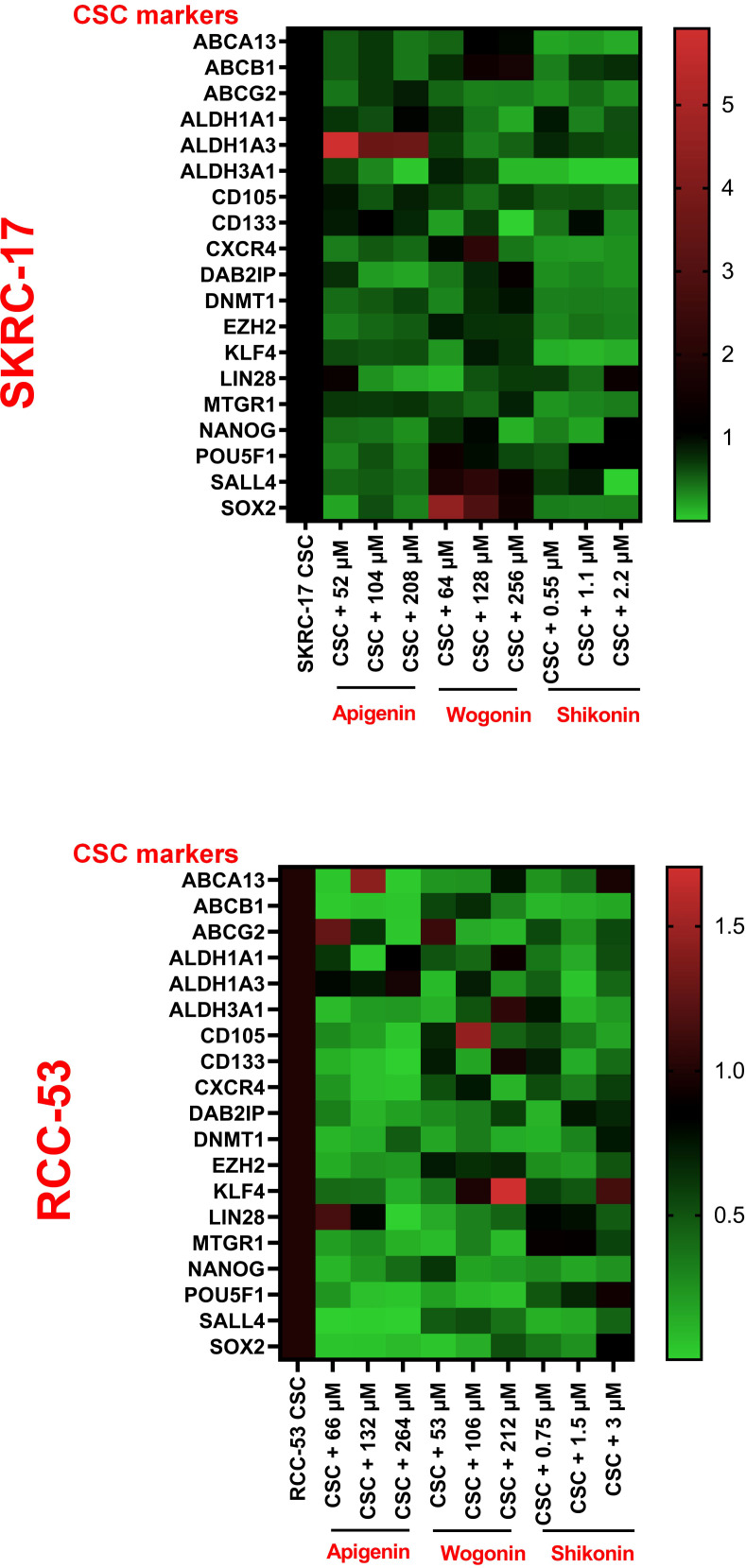
The impact of PTCs on the expression of CSC biomarkers. Analyzing the expression of different CSC biomarkers by RT-qPCR following treatment of RCC-53 and SKRC-17 with apigenin, wogonin, and shikonin. Results are shown as heatmaps. Normalized expression levels are displayed (n = 3).

To further determine the therapeutic strategies against CSC, 19 potential RCC CSC markers were selected. The CSCs of RCC-53 and SKCR-17 were treated with apigenin, wogonin, and shikonin in a concentration of 0.5 × IC50, 1 × IC50, and 2 × IC50. The mRNA expression was tested by RT-qPCR. Based on the results, apigenin showed downregulation of most of the markers in RCC-53 CSCs. Shikonin showed downregulation of most of the markers in SKRC-17 CSCs (**Figure 3**).

### 3.4 The impact of the combination ICI and shikonin on T cell subpopulations co-cultured with treated RCC adherent cells or CSCs

**Figure 4:**
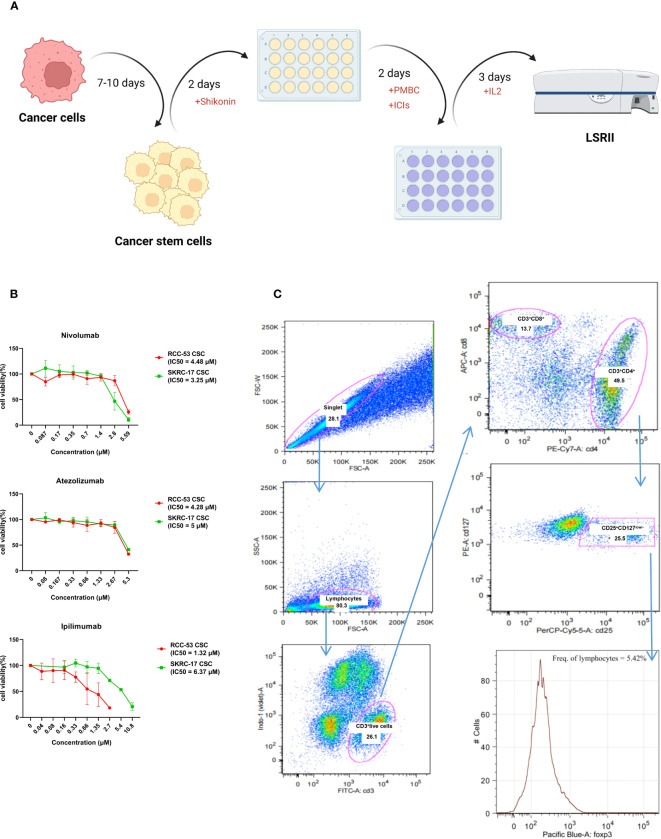
Workflow and gating strategy for T cell subpopulations in peripheral blood. **(A)** Workflow of the MLTC system. **(B)** The estimation of the half-maximal inhibitory concentration (IC50) of ICIs was performed using the CellTiter-Blue Cell Viability Assay and calculated by the logit regression model. **(C)** Representative plots showing the gating strategy for Treg analysis. Percentages of FoxP3^+^CD25^+^CD127^low/-^ lymphocytes among the CD4^+^T cells.

**Figure 5:**
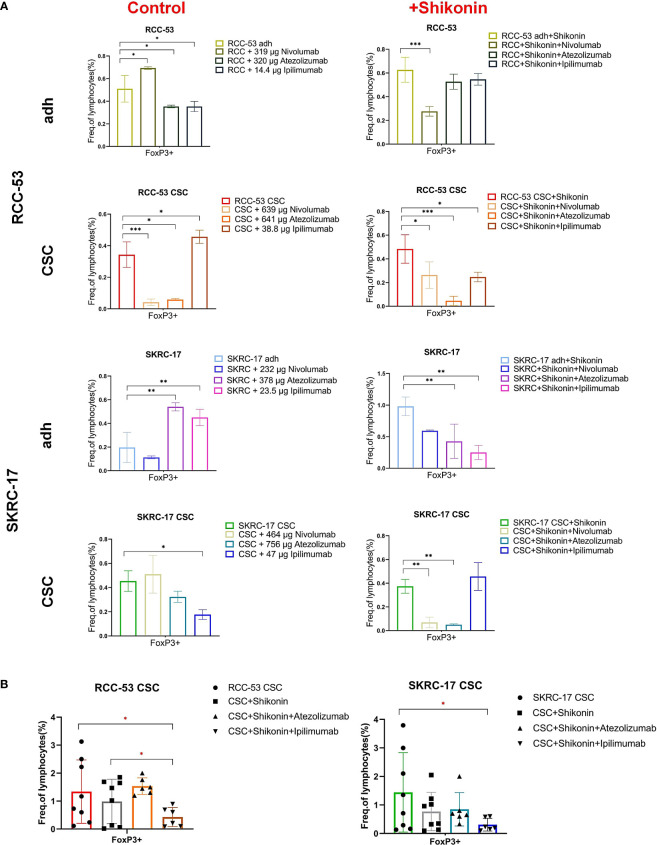
The impact of the combination treatment ICIs with or without shikonin on T cell subpopulations in PBMC co-cultured with RCC adherent cells or CSCs. The CD4^+^ CD25^+^ CD127^low/-^ FOXP3^+^ T cell subpopulations correspond to the effector Treg cells. **(A)** The phenotype of PBMCs of healthy donors after co-culture with treated RCC adherent cells or CSCs (nivolumab, atezolizumab, ipilimumab with or without shikonin, n = 3). **(B)** The phenotype of PBMCs from RCC patients co-cultured with CSCs after treatment with atezolizumab or ipilimumab with or without shikonin (n = 6, * *p* < 0.05, ** *p* < 0.01, *** *p* < 0.001, **** *p* < 0.0001).

Three ICIs (nivolumab, atezolizumab and ipilimumab) were selected to analyze the phenotype of PBMC in healthy donors co-cultured with RCC adherent cells or CSCs after treatment with or without shikonin. To guide the selection of treatment concentrations for ICIs, the cell viability was performed to determine the respective IC50 values of ICIs as shown in **Figure 4B**. The co-culture system is shown in **Figure 4A** and the gating strategy for the T cell subpopulations is shown in **Figure 4C**. The CD4^+^, CD25^+^, CD127^low^, FOXP3^+^ T cell subpopulation corresponds to the effector Treg cells. Based on the results, the percentage of the FoxP3^+^ Treg subpopulation was significantly decreased in the ICI group as compared to the group without treatment. Ipilimumab in SKRC-17 CSCs and RCC-53, atezolizumab in RCC-53 and RCC-53 CSCs as well as nivolumab in RCC-53 CSCs. After combination with shikonin, the percentage of the FoxP3^+^ Treg subpopulation was significantly decreased compared to the group without treatment in following groups: ipilimumab in SKRC-17 adherent cells and RCC-53 CSC, atezolizumab in SKRC-17 adherent cells, SKRC-17 CSCs, and RCC-53 CSCs, nivolumab in RCC-53 adherent cells, RCC-53 CSCs as well as in SKRC-17 CSCs (**Figure 5A**).

**Figure 6:**
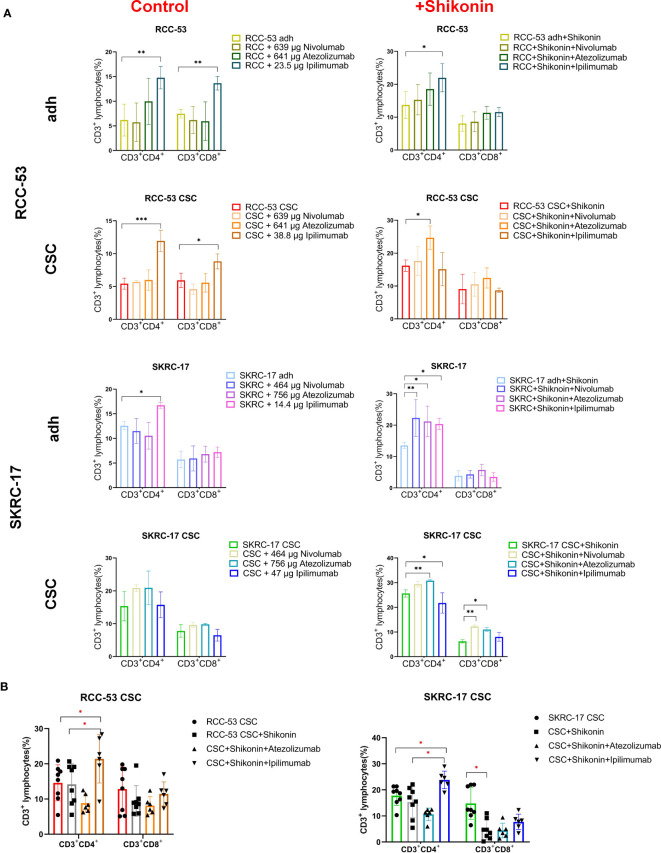
The impact of the combination treatment ICIs with or without shikonin on T-cell subpopulations in PBMC co-cultured with RCC adherent cells or CSCs. The CD3^+^CD4^+^ and CD3^+^CD8^+^ T cell subpopulations correspond to the helper T cells and cytotoxic T cells, respectively. **(A)** The phenotype of PBMCs of healthy donors after co-culture with treated RCC adherent cells or CSCs (nivolumab, atezolizumab, ipilimumab with or without shikonin, n = 3). **(B)** The phenotype of PBMCs from RCC patients co-cultured with CSCs after treatment with atezolizumab or ipilimumab with or without shikonin (n = 6, *p < 0.05, **p < 0.01, ***p < 0.001, ****p < 0.0001).

Moreover, ipilimumab significantly enhanced the CD3^+^CD4^+^ T cells in adherent cell lines of SKRC-17 and RCC-53 as well as in RCC-53 CSCs. Also the CD3^+^CD8^+^ T cells in RCC-53 adherent cells and RCC-53 CSCs were enhanced. In combination with shikonin, ipilimumab significantly enhanced CD3^+^CD4^+^ T cells in adherent cell lines of SKRC-17 and RCC-53, atezolizumab significantly elevated the CD3^+^CD4^+^ T cells population in SKRC-17 adherent cells and its CSCs as well as in RCC-53 CSCs. The CD3^+^CD8^+^ T cell population was enhanced by atezolizumab in SKRC-17 CSCs, and by nivolumab in SKRC-17 CSCs (**Figure 6A**).

Although the results in other groups may not exhibit consistent trends, the focus on the CSCs group provides valuable insights for determining the next steps in the study and guiding further experiments. So, atezolizumab and ipilimumab were selected in combination with shikonin for further testing in MLTCs with PBMC from RCC patients and CSCs from SKRC-17 and RCC-53. Ipilimumab again suppressed the FoxP3^+^ Treg subpopulation and enhanced the CD3^+^CD4^+^ T cells. Furthermore, compared with the single shikonin treatment group, ipilimumab combined with shikonin significantly decreased the FoxP3^+^ Treg subpopulation in RCC-53 and enhanced the CD3^+^CD4^+^ T cells population in SKRC-17 and RCC-53 (**Figures 5B**, **6B**). Based on this result, the selection of ipilimumab guided the subsequent steps of our study.

### 3.5 Identification of potential immunotherapeutic targets

**Figure 7:**
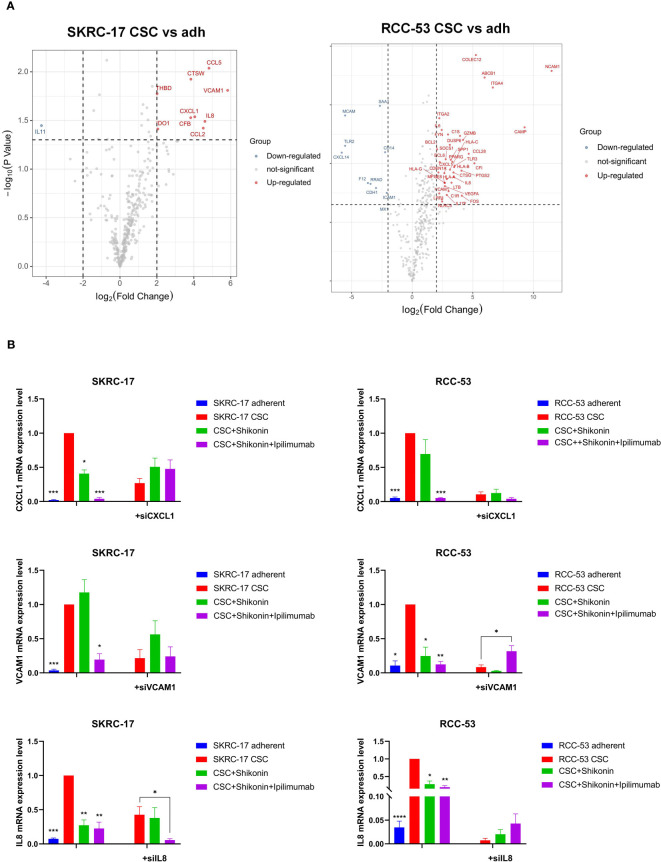
Identification of the potential immunotherapeutic targets for the combination treatment ipilimumab and shikonin. **(A)** Nanostring analysis was used to find differently expressed genes in CSCs versus adherent cell lines. **(B)** The expression of three potential immunotherapeutic targets was identified by RT-qPCR and siRNA transfection in adherent and CSC cell lines following treatment with ipilimumab +/-shikonin. (*p < 0.05, **p < 0.01, ***p < 0.001, ****p < 0.0001).

To further understand the relative immunotherapeutic targets and novel potential CSC markers in RCC, a NanoString analysis was done using the adherent cell lines and their corresponding CSCs without treatment, with treatment with shikonin alone as well as with shikonin combined with ipilimumab.Three common new CSC markers (IL-8, CXCL1 and VCAM1) were identified as promising immunotherapeutic targets due to the fact, that they were significantly higher expressed in each treatment group (**Figure 7A**). RT-qPCR analysis confirmed the higher expression of these three markers in RCC-53 CSCs and SKRC-17 CSCs compared to the adherent cell lines (**Figure 7B**). Furthermore, the combined treatment with shikonin and ipilimumab significantly inhibits the mRNA expression of *IL-8*, *CXCL1*, and *VCAM1*, verified by siRNA technology (**Figure 7B**). Moreover, the potential signaling pathways regulated by the different treatments groups were also analyzed using the NanoString analysis system: immune-related signaling pathway, cell cycle, macrophage functions and senescence pathways in RCC-53 CSC cell lines; NK cell functions, B-cell functions, interleukin pathways in SKRC-17 CSC cell lines (**Supplementary Figure S4**).

### 3.6 Network of potential target genes between combination treatment and *IL-8*, *CXCL1* and *VCAM1*

**Figure 8:**
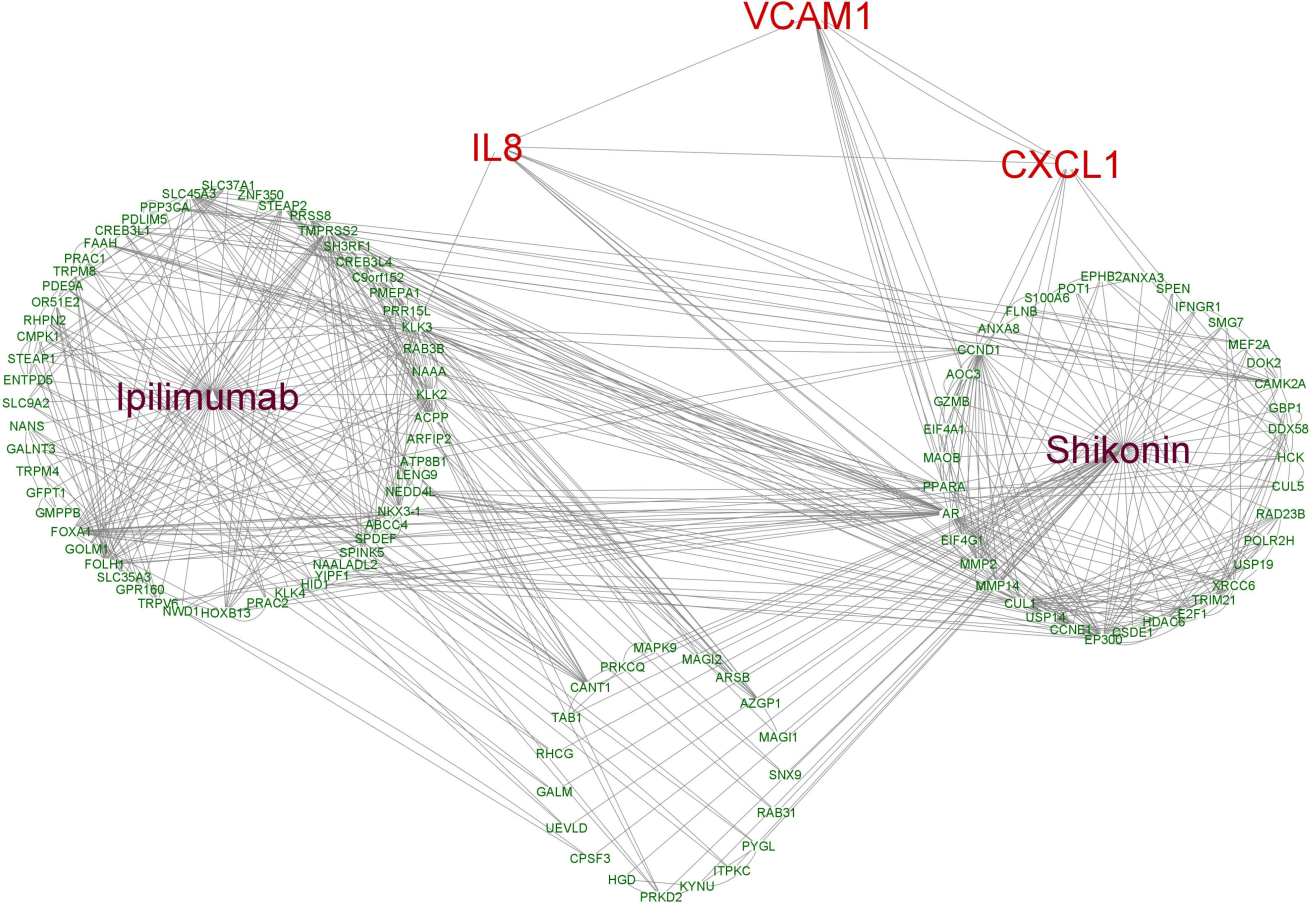
The interaction network based on the potential targets of ipilimumab and shikonin as well as *VCAM1*, *CXCL1* and *IL8*.

To further uncover the potential pharmacological mechanisms of various treatments, *IL-8*, *CXCL1* and *VCAM1* as well as predicted target genes of ipilimumab and shikonin were analyzed in networks. A total of 38 genes were predicted to be direct targets of shikonin and 55 genes were predicted to be targets of ipilimumab, of which 19 genes were found that directly target shikonin and have a connection with ipilimumab’s target genes. Interactions between the potential target genes and *IL-8*, *CXCL1* and *VCAM1* are shown in **Figure 8**. *IL-8*, *CXCL1*, and *VCAM1* are connected with each other, *IL-8* is connected with ipilimumab and shikonin potential target genes, and *CXCL1* and *VCAM1* are connected with shikonin potential target genes.

### 3.7 VCAM1 expression in ccRCC patients

**Figure 9:**
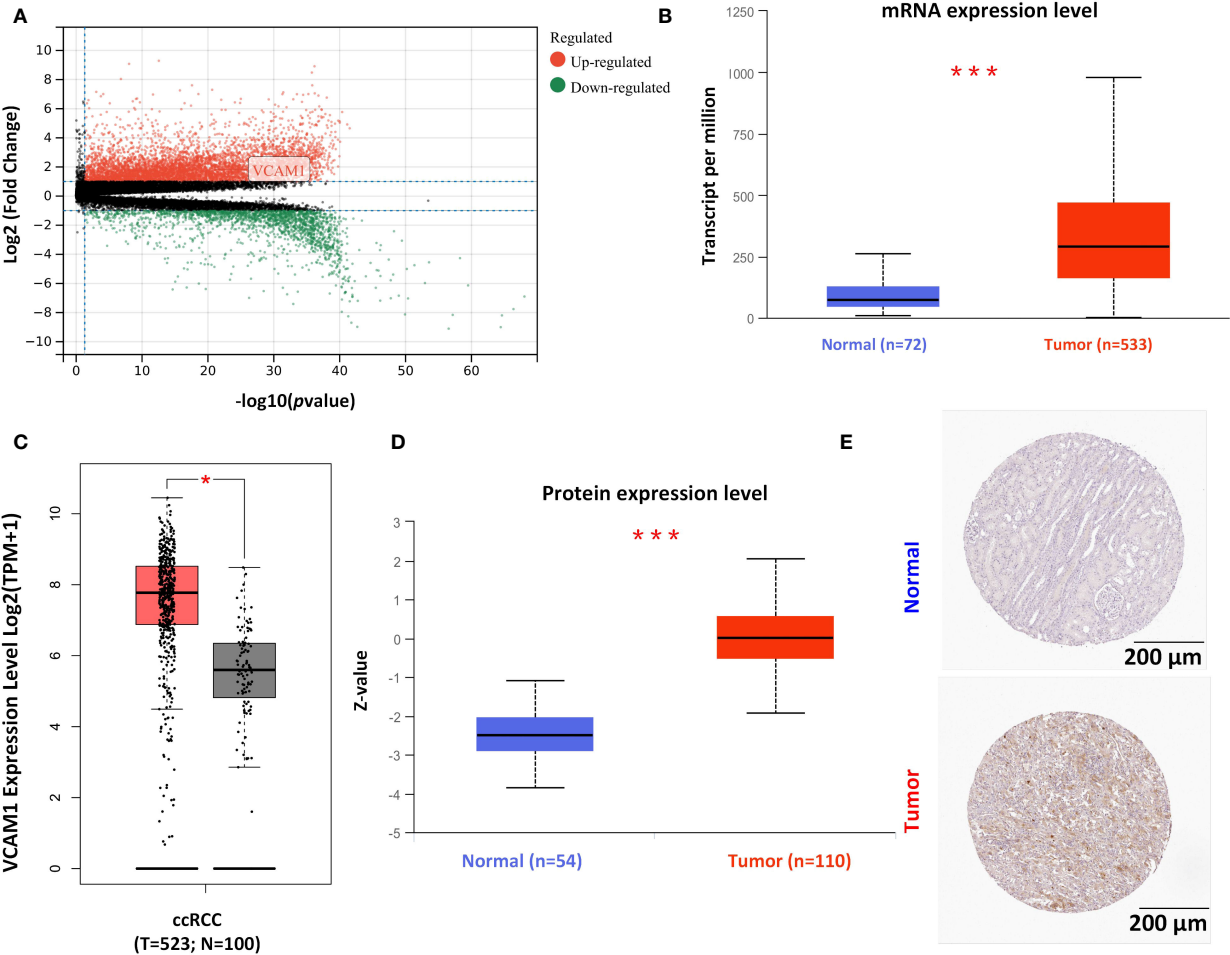
The expression pattern of *VCAM1* in RCC. **(A)** The volcano plot of differently expressed genes in ccRCC patients’ samples. **(B)** Comparison of the mRNA expression of *VCAM1* between tumor tissue (red) and normal tissue (blue) in RCC (***p <0.001) from The Cancer Genome Atlas (TCGA) project. **(C)** The normal tissue data from the Genotype-Tissue Expression (GTEx) database as controls compared with the corresponding data from the TCGA project. The results are presented as a box plot (*p < 0.05). Tumor samples in red and normal samples in black. **(D)** The comparison of VCAM1 protein expression between tumor tissue (red) and normal tissue (blue) in RCC (***p < 0.001). **(E)** VCAM1 protein expression was shown in immunohistological sections of normal and tumor renal tissue, obtained from the Human Protein Atlas database.

After identifying VCAM1 as a promising immunotherapeutic target in RCC through NanoString analysis, further detailed analyses were performed to investigate its potential in the treatment of RCC. All significantly differently expressed genes in ccRCC are shown in **Figure 9A**, of which *VCAM1* is highly expressed in the ccRCC data set. Significant expression differences of VCAM1 on the mRNA (**Figure 9B**) and protein level (**Figure 9D**) between tumor and normal tissues were found in ccRCC patients. Because not enough normal tissue samples were available in the TCGA database, normal tissues from the GTEx data set were used as control in **Figure 9C**. Moreover, the VCAM1 protein expression between normal samples and kidney cancer samples was further validated using The Human Protein Atlas. An example is shown in **Figure 9E**.

### 3.8 Correlation of VCAM1 expression with the survival and clinical characteristics of ccRCC patients

**Figure 10:**
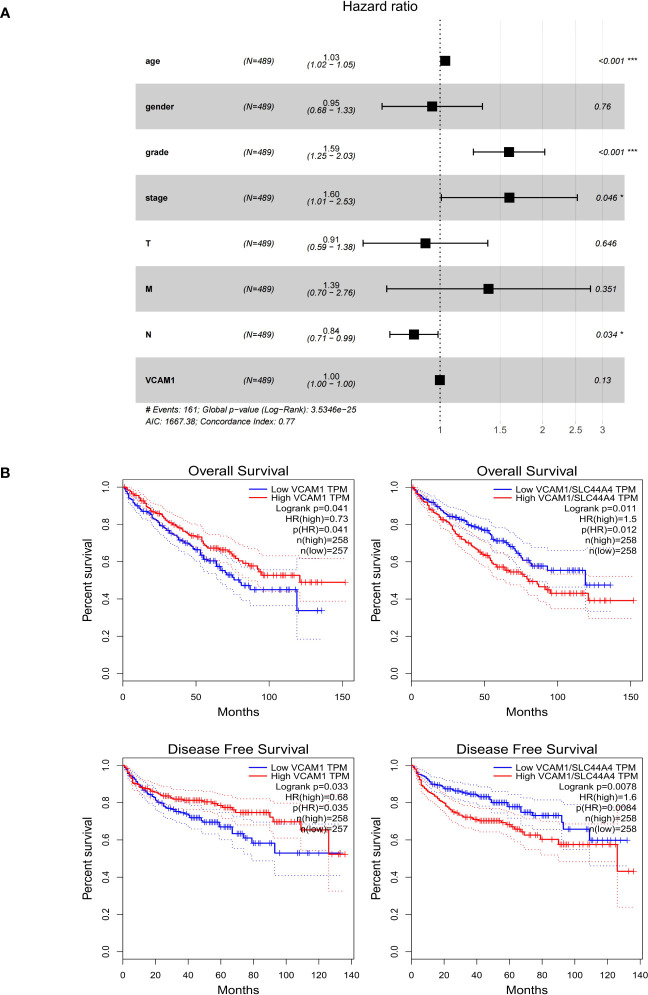
The prognostic value of *VCAM1* in RCC. **(A)** The multivariate cox regression analysis of the risk score, age, gender, grade, and TNM stage was used to evaluate the independent prognostic value of *VCAM1*. *p < 0.05, ***p < 0.001. **(B)** Correlation between *VCAM1* or *VCAM1/CTLA-4* gene expression, overall survival, and disease-free survival in ccRCC.

The clinical characteristics of ccRCC patients including stage, grade and TNM classification, gender and age were grouped into *VCAM1* high and low expression according to the median expression level. Multivariable Cox regression analysis showed that several clinical characteristics including age, grade, stage and N classification could be an independent prognostic factor to assess outcomes for ccRCC patients (**Figure 10A**). Based on the log-rank test in GEPIA, high mRNA expression of *VCAM1* (*p* = 0.041) was significantly associated with a better prognosis in ccRCC patients, as shown for overall survival (**Figure 10B**). In addition, after normalization by the *CTLA-4* (cytotoxic T-lymphocyte-associated Protein 4) gene expression (= ipilimumab target protein), lower expression of *VCAM1* mRNA was significantly associated with better prognosis in ccRCC patients (*p* = 0.012). For disease free survival, high expression of *VCAM1* mRNA) was significantly associated with better prognosis of ccRCC patients (*p* = 0.035). After normalization by the CTLA-4 gene expression, low *VCAM1* mRNA expression was significantly associated with better prognosis in ccRCC patients (*p* = 0.008) (**Figure 10B**).

### 3.9 Correlation of *VCAM1* expression with immune signatures in ccRCC patients

**Figure 11:**
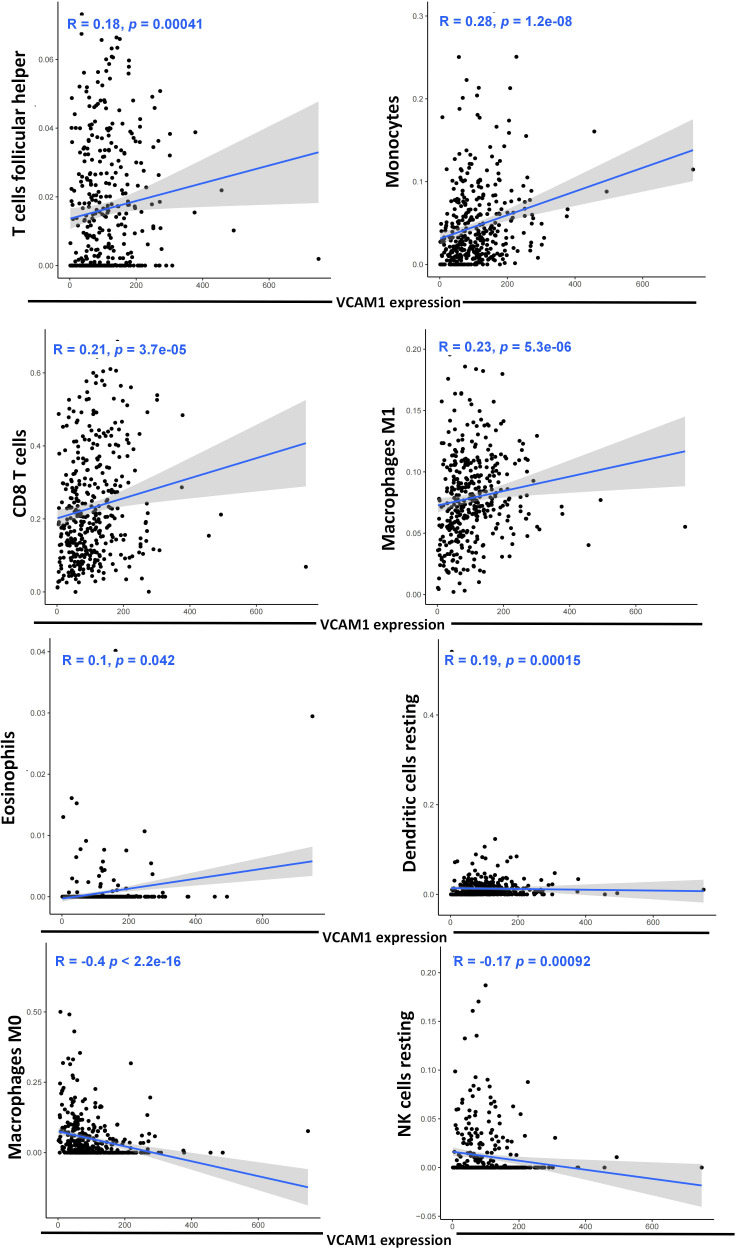
Correlation of eight TIC subpopulations with the *VCAM1* expression. The blue line in each plot shows the fitted linear model indicating the proportion tropism of the immune cell along with the *VCAM1* expression, and the Pearson coefficient was used for the correlation test.

**Figure 12:**
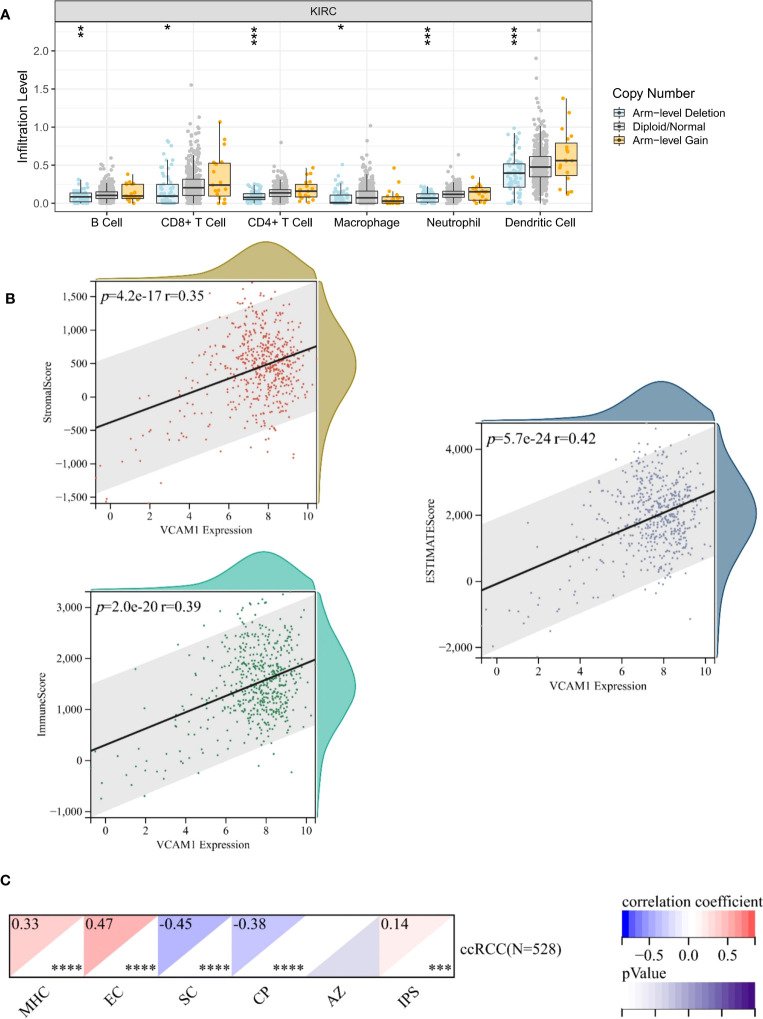
Correlation of *VCAM1* expression with the immune signatures. **(A)** *VCAM1* Copy number variant (CNV) affects the infiltrating levels of CD8^+^T cells, macrophages, neutrophils, and dendritic cells in ccRCC patients. **(B)** The correlation of the *VCAM1* expression and the ESTIMATEScore, the StromalScore and the ImmuneScore in ccRCC. **(C)** The correlation of the *VCAM1* expression and immunophenoscore (IPS) in ccRCC, MHC (Antigen processing), CP (checkpoint), EC (effector cells), SC (suppressor cells) and average Z-score (AZ). *p < 0.05, **p < 0.01, ***p < 0.001.

To explore a potential correlation between *VCAM1* expression and the ccRCC tumor microenvironment (TME), the proportion of tumor-infiltrating immune cell (TIC) subsets was determined, and eight types of TICs had different frequencies in *VCAM1* high versus low tumors. Follicular helper T cells, monocytes, CD8 T cells, M1 macrophages, eosinophils, and resting dendritic cells were more prevalent in *VCAM1* high tumors than in *VCAM1* low tumors, while M0 macrophages and resting NK cells were more abundant in *VCAM1* low tumors (**Figure 11**). Particularly, the copy number variants (CNV) of *VCAM1* CNV showed significant correlations with the infiltrating of B cells, CD8^+^ T cells, CD4^+^ T cells, macrophages, neutrophils, and dendritic cells (**Figure 12A**). Moreover, based on the TCGA plus GTEx database, significant correlations were found between *VCAM1* expression and the StromalScore, ESTIMATEScore, and ImmuneScore (**Figure 12B**). In 2017, Charoentong et al. developed an algorithm named immunophenoscore (IPS), which can predict the efficiency of anti- CTLA-4 and anti-PD-1 antibody therapies including MHC (Antigen processing), CP (checkpoint), EC (effector cells), and SC (suppressor cells) (28). We evaluated the differences in MHC, EC, SC, CP, ICS and average Z-score (AZ) with *VCAM1* expression. *VCAM1* expression was significantly positively correlated with the MHC, EC, and immunophenoscore (IPS), and negatively correlated with the SC and CP scores (**Figure 12C**).

### 3.10 Correlation of *VCAM1* expression with associated genes and pathways

**Figure 13:**
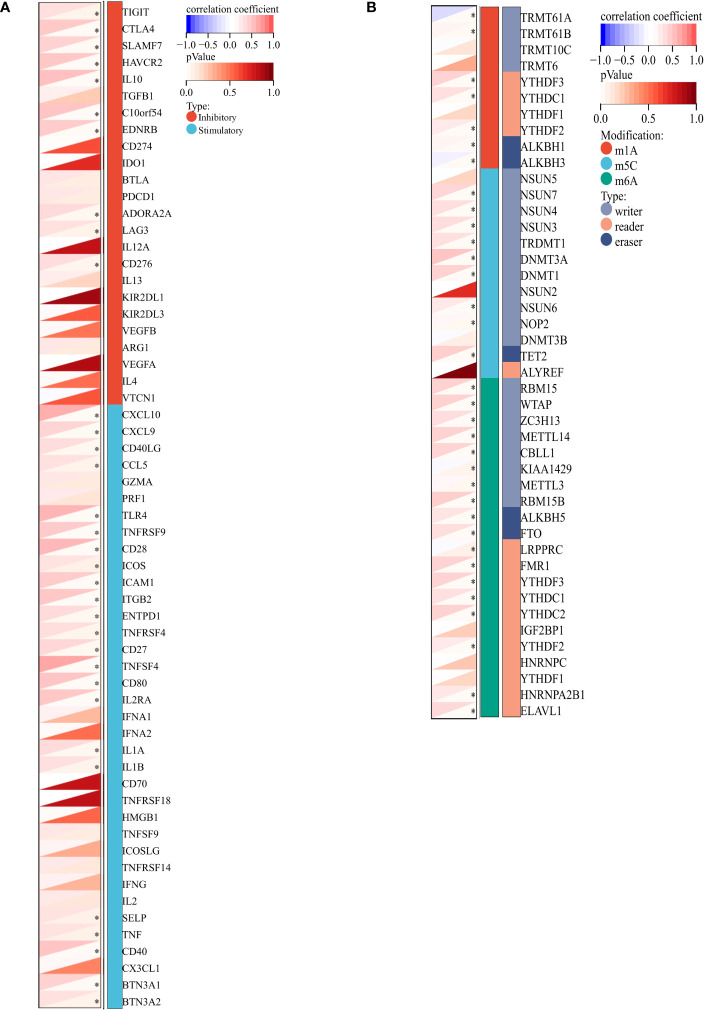
*VCAM1*-related genes in ccRCC. **(A)** Co-expression analysis between 60 immune-related genes (inhibitory: 24 and stimulatory: 36) and *VCAM1* gene in the ccRCC data set. **(B)** Co-expression analysis between 44 RNA modifications modulate genes (N1-methyladenosine (m1A), 5-methylcytosine (m5C), N6-methyladenosine (m6A) and *VCAM1* gene in the ccRCC data set. *p < 0.05.

**Figure 14:**
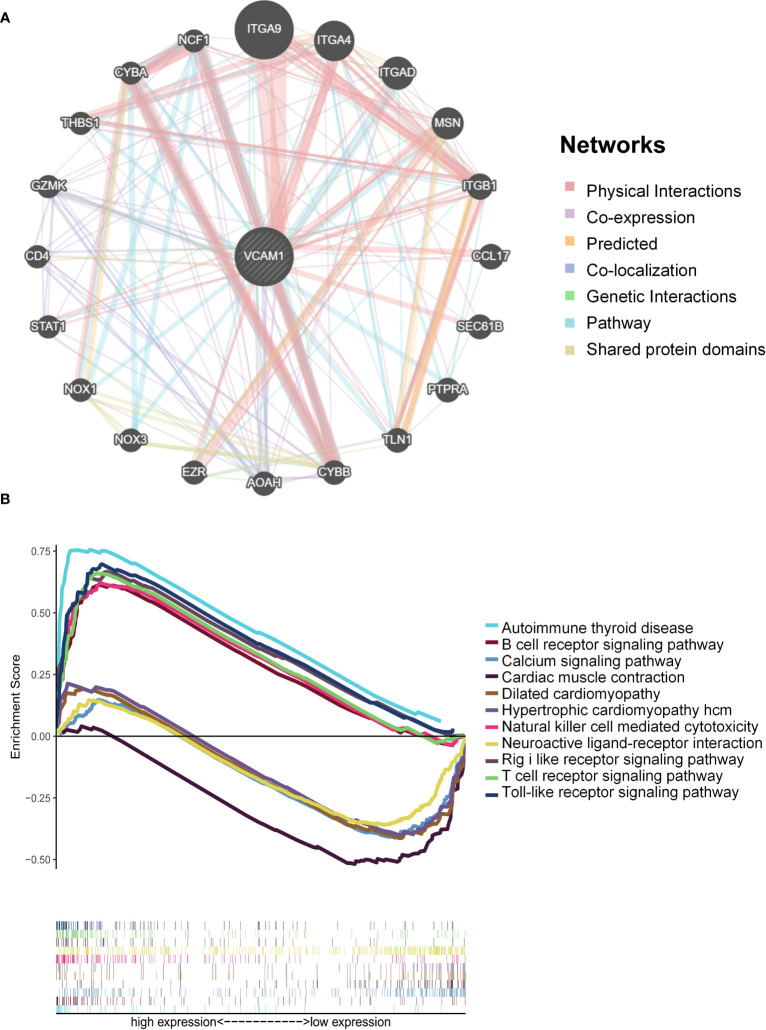
VCAM1-related pathways enrichment analysis. **(A)** Protein-protein interaction (PPI) network for the top 20 VCAM1-related proteins based on the GeneMANIA online tool. Different colors of the network edges indicate the bioinformatic methods applied: physical interaction, co-expression, predicted, co-localization, pathway, genetic interaction, and shared protein domains. **(B)** KEGG functional annotation of the *VCAM1* gene in ccRCC via GSEA analysis. Peaks of curves upward indicate positive regulation and peaks of curves downward represent negative regulation. Differently colored curves indicate that the *VCAM1* gene regulates different functions or pathways.

The relationship between *VCAM1* gene expression and a total of 60 immune checkpoint genes (inhibitory (24) and stimulatory (29)) and 44 RNA modifications modulate genes (N1-methyladenosine (m1A) (10), 5-methylcytosine (m5C) (13) and N 6-methyladenosine (m6A) (21) in ccRCC patients was analyzed (**Figures 13A, B**). We found that *VCAM1* expression showed a positive correlation with several immune checkpoint genes, for example *CTLA4*, *HAVCR2*, *IL10*, *CXCL9* and *CXCL10*. Thirty-four RNA modifications modulate genes were significantly correlated with *VCAM1* expression. Furthermore, the top 20 *VCAM1*-related genes from the GeneMANIA online tool were analyzed by the protein-protein interaction (PPI) network in **Figure 14A**. Finally, we used GSEA that included related genes in human to find general enrichment trends and to identify KEGG enrichment of different expression levels of *VCAM1* in ccRCC patients (**Figure 14B**). Autoimmune thyroid disease, B cell receptor signaling pathway, natural killer cell mediated cytotoxicity, rig I like receptor signaling pathway, T cell receptor signaling pathway, toll-like receptor signaling pathway were enriched in the high *VCAM1* expression group. Calcium signaling pathway, cardiac muscle contraction, dilated cardiomyopathy, hypertrophic cardiomyopathy (HCM), and neuroactive ligand-receptor interaction were enriched in the low expression group.

## 4 Discussion

Over 30% of ccRCC patients already have distant metastases at the time of initial diagnosis and most of them are not sensitive to chemotherapy and radiation therapy. Currently, angiogenesis inhibition with TKIs combined with the immune checkpoint inhibitor has revolutionized the treatment landscape of ccRCC patients with metastases. However, higher objective response rate and survival rates were seen in immune checkpoint positive patients and those in the intermediate-poor risk subgroups of the International mRCC Database Consortium (IMDC). Thus, additional therapies or promising additives to the current therapeutic strategies for those non-responders are urgently needed (1, 2). The presence of CSCs was discussed to be one of the causes for resistance to standard treatment strategy due to its strong abilities of self-renewal and differentiation (10, 13). Moreover, to target CSCs, some phytochemicals had a high potential to uncover the molecular mechanisms of metastatic initiation and dynamics of RCC CSCs. Thus, the combination therapy strategy between phytochemicals and ICIs was came up to improve the therapy management of RCC in this study. The key findings can be summarized as follows: (1) Shikonin, wogonin, and apigenin were promising phytochemicals for targeting the RCC CSCs among 11 phytochemicals tested, which showed a substantially higher binding probability prediction score and more intersection genes with ccRCC immune-related genes, respectively. (2) Shikonin was the most promising PTC, which can significantly inhibit the ability of migration and invasion as well as increasing apoptosis of CSCs. (3) Among the three ICIs tested, the combination treatment of shikonin with ipilimumab confirmed a significantly decrease of the frequency of CD4^+^ CD25^+^ CD127^low/-^ FOXP3^+^ Treg in PBMC of ccRCC patients as well as an enhancement of the CD3^+^CD4^+^ T cells, compared to the group without treatment. (4) VCAM1, CXCL1 and IL8 were identified as novel CSC markers that are strongly related to the combination therapy strategy. (5) The expression of VCAM1 was statistically higher both at the mRNA and protein level, and was significantly correlated with the clinical characteristics and several immune-related signatures in ccRCC patients.

Based on our previous research, the influence of shikonin depends on the RCC cell line being investigated. For example, shikonin was proven to significantly trigger necrosis and apoptosis as well as enhances autophagy via the elevation of ROS (reactive oxygen species) level and p38 activity in different RCC cell lines like Caki-1 and ACHN cells in proportion to its concentration (29). Consistently, recent research in 2022 showed that shikonin has a strong ability to induce apoptosis and necroptosis of parental and sunitinib-resistant RCC cell lines (30). These findings suggested that shikonin may be an additional option for the treatment of patients with advanced and therapy resistant RCC. More recently, a new study conducted in 2023 showed that shikonin significantly reduces PD-L1 expression specifically on macrophages, without affecting PD-1 expression on T cells, both *in vivo* and *in vitro*. Furthermore, the study revealed that shikonin’s mechanism of action involves the attenuation of PD-L1 expression on macrophages by downregulating phosphorylation and nuclear import of PKM2 (pyruvate kinase M2), which was found to bind to the PD-L1 promoter, thereby influencing its expression (31). Unfortunately, so far not so many combination treatment studies between shikonin and immune therapy were explored. In 2019, Huang et al. found that the synergistic combination of shikonin and the suppressor of PD-L1 (JQ1) as well as the treatment potency of the PD-L1 checkpoint blockage mannosylated lactoferrin nanoparticulate system could reprogram the tumor immune microenvironment and metabolism via tumor-associated macrophages and glucose metabolism (32). This result revealed that the poly-pharmacological activities of shikonin were ideal for a combined immunotherapeutic application. Particularly, the suppressive effect of glucose metabolism by shikonin leveraged a positive regulation of the cancer-immunity circle. More and more combination strategies with phytochemicals and immune checkpoint inhibitors were investigated and demonstrated an effective strategy for cancer immunotherapy to date (33). For example, the combination of curcumin and anti-CTLA-4 therapy enhanced the anti-tumor effects via inhibition of PD-L1 and COP9 signalosome 5 compared to the single treatment group (34). Icaritin plus anti-PD-1/CTLA-4 treatment reduced the growth of melanoma cell line in C57BL/6 mice by 65% compared to anti-PD-1/CTLA-4 treatment alone (34.2%) (35). Collectively, phytochemicals showing anti-tumor effects in combination with immune checkpoint inhibitors are a promising therapeutic option for clinical trials in the future.

Immune recognition of therapeutic targets is essential for the immune response against tumors, also avoiding severe adverse effects. VCAM1, IL8 and CXCL1 were identified in ipilimumab and shikonin treatment as potential immunotherapeutic targets and as novel RCC CSC markers in this study. Similarly, the concentration of these factors were altered via ipilimumab plus bevacizumab treatment in patients with metastatic melanoma (36), which confirms the function as immunotherapeutic targets in ICI combination therapy. VCAM1 as one of adhesion molecules contributes to critical physiologic functional roles in cancer metastasis and therapy resistance (37), and is the only one showing a significantly higher expression level in RCC tissues than in normal tissues both at protein and mRNA level. Currently, the specific functional role of VCAM1 in RCC is less explored, with data pointing towards its overexpression involving in RCC tumor immune evasion (38). In 2022, a novel specific cell surface expression pattern in RCC represented by the NCI-60 tumor cell panel was identified and confirmed that VCAM1 is a promising novel immunotherapeutic target for the treatment of renal cancer particularly (39). Moreover, our results are consistent with the previous report that VCAM1 plays a protective role in RCC as a prognostic biomarker (40). However, this tendency of prognostic result is reversed following normalization by the CTLA-4 genes expression (**Figure 10B**), implying that the combination therapy strategy may have multiple factors that could influence the prognostic progress.

In this study, we hypothesize that shikonin may be a beneficial combination partner for ipilimumab for the treatment of ccRCC patients due to its strong inhibitory effect on cancer stem cells, the significant reduction of FoxP3^+^ Treg cells in PBMC of patients and the activation of the endogenous effector CD3^+^CD8^+^ and CD3^+^CD4^+^ T cells in response to the recognition of tumor specific antigens. Despite the numerous limitations encountered in this study, such as the unavailability of PBMC samples for testing certain therapeutic settings and the assessment of off-target effects in cell cytotoxicity about PTCs, it is important to note that this study represents one of the pioneering efforts in the field. In future studies, efforts will be made to overcome these limitations by expanding sample availability and incorporating a wider range of therapeutic settings, allowing for a more comprehensive understanding of the topic. In summary, we propose that a combination of shikonin and ipilimumab could be a promising treatment strategy.

## Data availability statement

The raw data supporting the conclusions of this article will be made available by the authors without undue reservation.

## Ethics statement

The studies involving human participants were reviewed and approved by Ethics Committee of the Ludwig Maximilians University Munich. The patients/participants provided their written informed consent to participate in this study.

## Author contributions

CL and HP contributed to conception and design of the study. CL and LW organized the database and performed the statistical analysis. CL wrote the first draft of the manuscript. BS, AB, and HP offered technical or material support, critical reading, and text revisions. All authors contributed to the article and approved the submitted version.
