# Supplementary material for: Identification of a novel combination treatment strategy in clear cell renal cell carcinoma stem cells with shikonin and ipilimumab
Source: Front Immunol. 2023 Aug 9;14:1186388. doi: 10.3389/fimmu.2023.1186388 (PMC10445237; doi:10.3389/fimmu.2023.1186388)
Supplement: Supplementary file 1 [file DataSheet_1.pdf]

*Supplementary Material*

# Identification of novel combination treatment strategy in clear cell renal cell carcinoma stem cells with shikonin and ipilimumab

**Chen Lyu[1], Birgit Stadlbauer[1,2], Lili Wang[1,3], Alexander Buchner[1,2], Heike Pohla[1,2*]**

[1]Tumor Immunology Laboratory, LIFE Center, LMU Klinikum, University Munich, Germany

[2]Department of Urology, LMU Klinikum, University Munich, Germany

[3]Department of Radiology, First Affiliated Hospital, Zhejiang University School of Medicine, Hangzhou, China

*** Correspondence:**

Heike Pohla

heike.pohla@med.uni-muenchen.de

**Supplementary Figures**

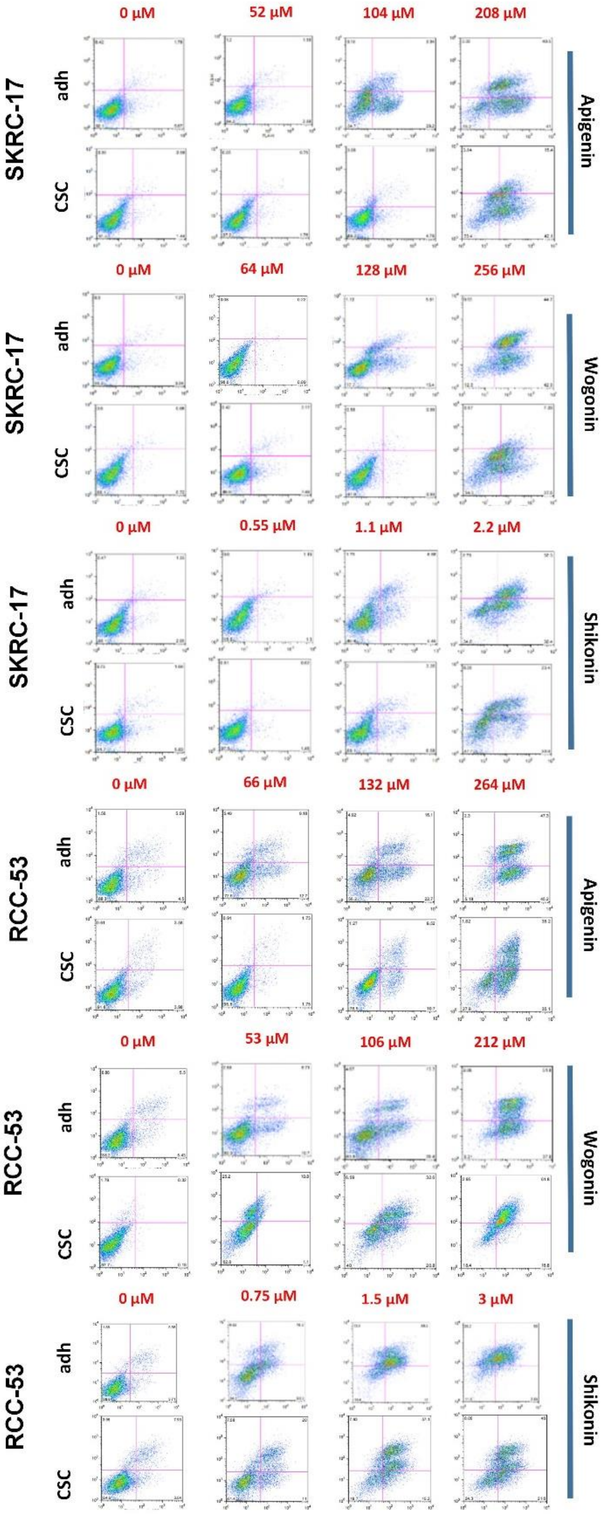


**Supplementary Figure S1. Apoptosis assay**. SKRC-17 adherent cell, SKRC-17 CSCs, RCC-53 adherent cells and RCC-53 CSCs were treated with apigenin, wogonin, and shikonin.

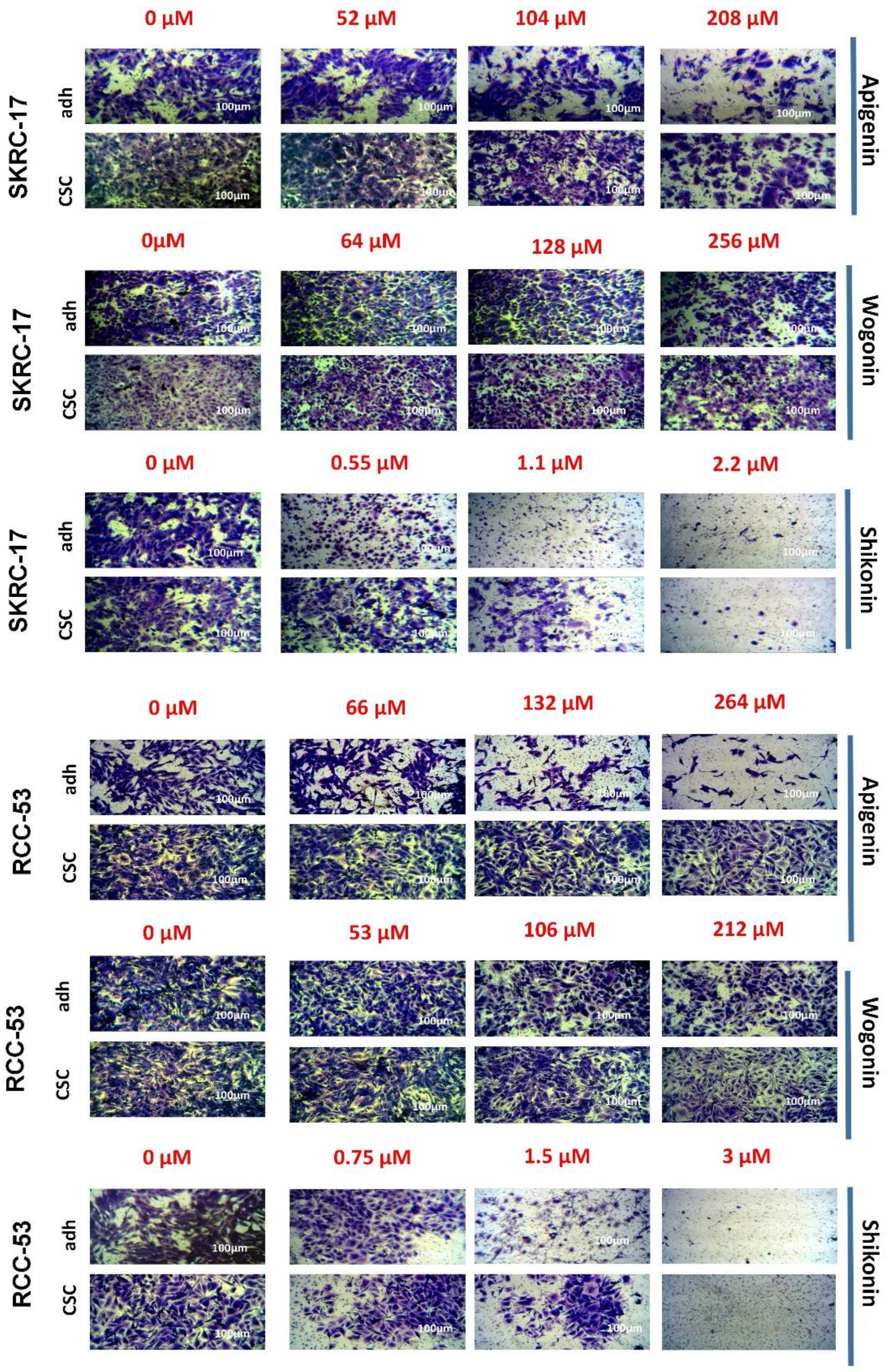


**Supplementary Figure S2. Invasion assay**. Photos were taken at the magnification of 40x and the cells were counted (three fields per insert; Fiji ImageJ software).

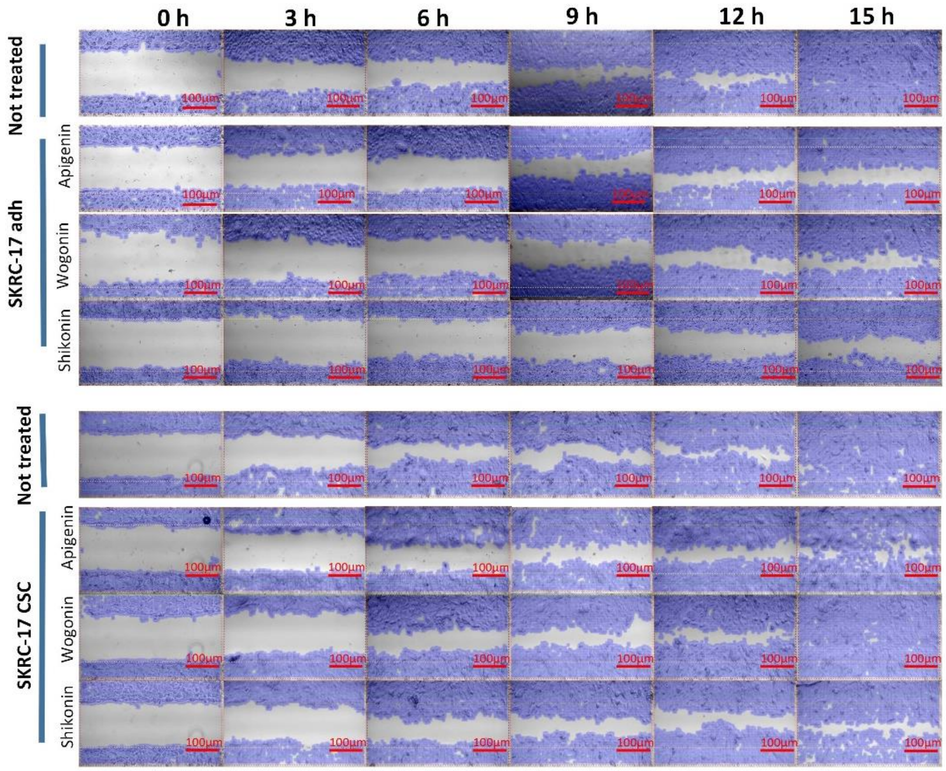


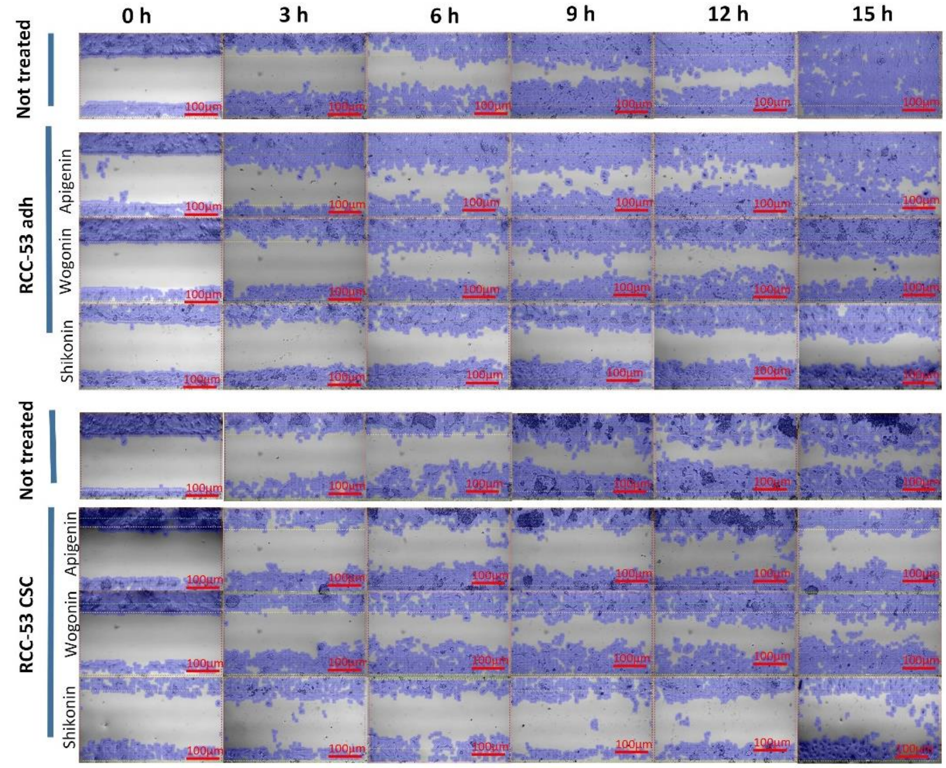


**Supplementary Figure S3. Migration assay.** Photos were taken under the microscope at different time points at the magnification of 40× (Bresser GmbH DE-46414 Rhede Germany).

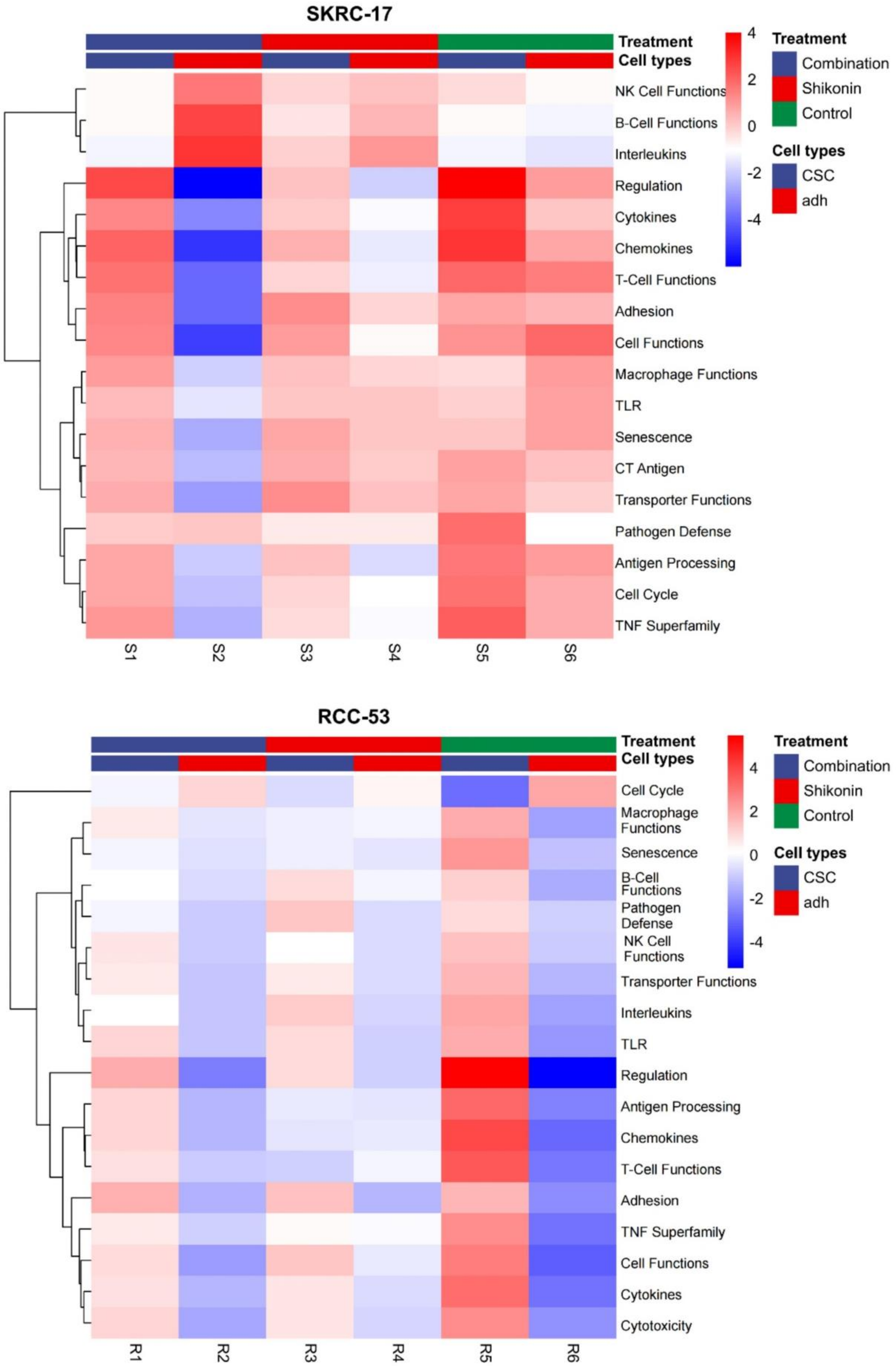


**Supplementary Figure S4. Heatmap of the differentially expressed pathways**. Between CSCs and adherent cells, groups were treated with or without shikonin or with the combination shikonin plus ipilimumab (NanoString analysis).
